# Histopathology of aortic complications in bicuspid aortic valve versus Marfan syndrome: relevance for therapy?

**DOI:** 10.1007/s00380-015-0703-z

**Published:** 2015-07-01

**Authors:** Nimrat Grewal, Romy Franken, Barbara J. M. Mulder, Marie-José Goumans, Johannes H. N. Lindeman, Monique R. M. Jongbloed, Marco C. DeRuiter, Robert J. M. Klautz, Ad J. J. C. Bogers, Robert E. Poelmann, Adriana C. Gittenberger-de Groot

**Affiliations:** Department of Cardiothoracic Surgery, Leiden University Medical Center, Leiden, The Netherlands; Department of Anatomy and Embryology, Leiden University Medical Center, Leiden, The Netherlands; Department of Cardiology, Academic Medical Center, Amsterdam, The Netherlands; Department of Molecular Cell Biology, Leiden University Medical Center, Leiden, The Netherlands; Department of Vascular Surgery, Leiden University Medical Center, Leiden, The Netherlands; Department of Cardiology, Leiden University Medical Center, Postal zone S-5-24, P.O. Box 9600, 2300 RC Leiden, The Netherlands; Department of Cardiothoracic Surgery and Heart Valve Bank, Erasmus University Medical Center, Rotterdam, The Netherlands; Department of Biology, Integrative Zoology, Leiden University Medical Center, Leiden, The Netherlands

**Keywords:** Aorta, Aneurysm, Immunohistochemistry, Molecular biology, Pathology

## Abstract

Patients with bicuspid aortic valve (BAV) and patients with Marfan syndrome (MFS) are more prone to develop aortic dilation and dissection compared to persons with a tricuspid aortic valve (TAV). To elucidate potential common and distinct pathways of clinical relevance, we compared the histopathological substrates of aortopathy. Ascending aortic wall biopsies were divided in five groups: BAV (*n* = 36) and TAV (*n* = 23) without and with dilation and non-dilated MFS (*n* = 8). General histologic features, apoptosis, the expression of markers for vascular smooth muscle cell (VSMC) maturation, markers predictive for ascending aortic dilation in BAV, and expression of fibrillin-1 were investigated. Both MFS and BAV showed an altered distribution and decreased fibrillin-1 expression in the aorta and a significantly lower level of differentiated VSMC markers. Interestingly, markers predictive for aortic dilation in BAV were not expressed in the MFS aorta. The aorta in MFS was similar to the aorta in dilated TAV with regard to the presence of medial degeneration and apoptosis, while other markers for degeneration and aging like inflammation and progerin expression were low in MFS, comparable to BAV. Both MFS and BAV aortas have immature VSMCs, while MFS and TAV patients have a similar increased rate of medial degeneration. However, the mechanism leading to apoptosis is expected to be different, being *fibrillin*-*1* mutation induced increased angiotensin-receptor-pathway signaling in MFS and cardiovascular aging and increased progerin in TAV. Our findings could explain why angiotensin inhibition is successful in MFS and less effective in TAV and BAV patients.

## Introduction

Bicuspid aortic valve (BAV) is the most common congenital cardiac malformation, with a prevalence of 1 % in the general population [1]. This anomaly is associated with complications as aortic stenosis and/or regurgitation as well as critical aortic dilation, with an increased risk of dissection and rupture. Aortic dilation is also a key feature of the clinical presentation in patients with Marfan syndrome (MFS). In MFS, mutations in the *fibrillin*-*1* gene, encoding for the fibrillin-1 protein, account for approximately 70–93 % of patients who meet the diagnostic criteria [2]. Due to the *fibrillin*-*1* mutation, the aorta in MFS exhibits markedly abnormal elastic properties which are assumed to lead to a decrease in compliance and progressive increase in dilation [3].

Both patients with MFS and BAV show aortic dilation but the anatomic site of vulnerability is distinct in both conditions. While maximal aortic dilation is observed above the sinotubular junction in BAV, in the MFS population, it is mainly found at the level of the sinuses of Valsalva, also referred to as aortic root [4].

To unravel the pathogenetic mechanism leading to aortic wall pathology in BAV, differences between the diseased aortic wall of patients with BAV and patients with a tricuspid aortic valve (TAV) were studied previously [5]. We found that the ascending aorta in BAV is intrinsically different from TAV patients. The vascular smooth muscle (VSMC) cell layer is less well differentiated in BAV, while inflammation and accelerated aging attribute to the aortic pathology in TAV [5].

Despite the VSMC immaturity, not all patients with BAV carry an increased risk for aortic dilation. In a previous study, we defined a panel of markers which could differentiate the non-dilated BAV patients, in a susceptible and non-susceptible subgroup for future dilation, being c-Kit, a marker for dedifferentiated VSMCs, and its phosphorylated state (pc-Kit) triggered by the presence of matrix metalloproteinase-9 (MMP9) influencing hypoxia-inducible-factor-1-alpha (HIF1α) and endothelial nitric oxide synthase (eNOS) [6].

The aortic wall in MFS and BAV has been described to have similarities, like an increased MMP activity and decreased fibrillin-1 expression [7, 8]. However, cytolytic necrosis, (also termed medial degeneration), defined as VSMC dropout, apoptosis, and elastic fiber degeneration, highly characteristic for MFS and the dilated aorta in TAV, are far less obvious in BAV [5, 7]. Although clinically, symptoms of aortic wall pathology in MFS and BAV overlap, still some striking differences remain less well understood. For instance, BAV patients rarely possess Marfanoid characteristics; conversely in patients with MFS, the risk of concomitant BAV syndrome is only slightly increased (4.7 %) [9]. Furthermore, the risk for aortic dilation in BAV, although higher than in the general population, remains low when compared to MFS where the majority of patients is prone to severe aortic wall disease. In MFS, moreover, not only aortic dilation is a critical aortic complication, but also dissections in a non-dilated aortic wall and at a younger age, as compared to the BAV, have been reported [10, 11]. In MFS furthermore, angiotensin-receptor-blockers (ARBs) as losartan have been identified as a potentially therapeutic agent to prevent progressive dilation of the ascending aorta [12–18]. A similar positive effect in preventing aortic complications, by reducing the angiotensin pathway signaling is, however, not seen in the BAV population [19]. It is therefore interesting to study which similarities are actually present immunohistochemically between the ascending aortic wall in both diseases and the dilated aortic wall in the TAV, despite possible different clinical sequelae in future. What factors cause increased weakness of the aortic wall in MFS and are these similar in BAV? Why is aortic wall pathology seen in BAV and MFS not comparable to the aortic dilation complications in patients with TAV?

To study this, we compared the aortic wall between MFS, BAV, and TAV, starting with the investigation of general histopathological features, including cytolytic necrosis, inflammation, elastin lamellae degradation, and VSMC apoptosis. The level of expression of fibrillin-1 was also studied in these groups. As VSMCs play a role in the synthesis and assembly of fibrillin-1, we compared our findings of fibrillin-1 expression to that of differentiation and maturation of the vascular wall, being the VSMC differentiation markers lamin A/C and progerin [5]. We further studied the expression of the pc-Kit pathway (c-Kit, pc-Kit, MMP9, HIF1α, and eNOS) to investigate whether these markers, indicative of BAV aortic dilation [6], are also applicable in the MFS group.

## Materials and methods

### Ethical statement

The institutional ethics committee at the Leiden University Medical Centre, Leiden approved this study. The Academic Medical Center (AMC), Amsterdam provided us with eight MFS biopsy aorta specimens, with approval of the Medical Ethical Committee. The Heart Valve Bank, Thoraxcenter, Erasmus Medical Center (EMC), Rotterdam, provided six non-dilated BAV aortic wall samples which were not suitable for transplantation, approved by their Scientific Advisory Board.

### Patients and tissue samples

Ascending aortic wall samples were collected from non-MFS individuals with TAV and BAV, with and without dilation. Based on the ACC/AHA guidelines, dilation was clinically defined by surpassing an ascending aortic wall diameter of 45 mm [20]. The study population was divided in five groups: (1) TAV without dilation, termed TA (*n* = 11, mean age 64.5 ± 9.0 years) obtained post mortem, (2) TAV with dilation, termed TAD (*n* = 12, mean age 72.3 ± 11.2 years) collected during elective replacement, (3) BAV without dilation, termed BA (*n* = 17, mean age 55.8 ± 9.8 years) representing a unique group from patients with stentless root replacement (the biopsy material was collected as residue waste material from the proximal anastomosis), and the 6 biopsies received from the EMC, (4) BAV with dilation, termed BAD (*n* = 19, mean age 60.7 ± 7.8 years), collected during elective replacement, and (5) MFS aortic wall without ascending aorta dilation, termed MFS (*n* = 8, mean age 34.2 ± 11.0 years), derived after elective replacement of the dilated root (the biopsy material was collected as residue waste material from the proximal anastomosis). Groups one to four have been reported previously [5, 6], and in the current study, additional staining with fibrillin-1 and apoptosis markers was performed for comparison with the newly described MFS group. In this study, we paid additional attention to the underlying aortic valve pathology of the study population. The TA group showed no valve pathology, and the TAD group showed variably either no valve pathology or aortic valve stenosis or regurgitation. In the non-dilated BAVs, six patients had a non-pathologic aortic valve; these were the specimens we received from the EMC, and the remainder of the BA group showed aortic valve stenosis, aortic valve regurgitation, or a combination of both. In the dilated BAV, group 3 patients had a non-pathologic aortic valve. All MFS patients had a non-pathologic valve and a dilated aortic root; specimens were hence obtained during a valve-sparing root replacement, which was performed in this patient group.

### Sample processing and routine histology

Specimens were sectioned and stained as described previously [5, 6]. Briefly, following excision, all specimens were fixed, decalcified, and paraffin embedded. Transverse sections (5 µm) of the paraffin-embedded tissue were deparaffinated and rehydrated after which they were stained with hematoxylin–eosin (HE) and resorcin fuchsin (RF) to study the morphology of the vessel wall. Aortic inflammation was quantified using the HE-stained sections, indexed from zero (no inflammatory cells) to 6 (large clusters of cells). In RF-stained sections, the maximum intimal thickness was quantified in µm and the organization of the elastic lamellae in the media was evaluated.

### Immunohistochemistry

Sections were stained following the protocol described previously [5, 6]. An overview of the primary and secondary antibodies used is given in Table 1.

**Table 1:** Immunohistochemistry reagents

Primary antibody	Vendor, order number	Concentration	Secondary antibody	Mechanism
Anti-αSMA	A2547, Sigma-Aldrich Chemie, Darmstadt, Germany	1:5000	RAM-PO (1:250) (DAKO p0260)	Smooth muscle cell differentiation
Anti-cleaved-caspase-3	9661, Cell Signaling, Beverly, USA	1:250	GAR (1:200) and NGS (1:66) (Vector Laboratories, USA, BA-1000 and S1000)	Apoptosis
Anti-SM22α	AB10135, Abcam, Cambridge, UK	1:100	GAR and NGS	Smooth muscle cell differentiation
Anti-smoothelin	16101, ProgenBiotechnik, Heidelberg, Germany	1:200	HAM (1:200) and NHS (1:66) (Vector Laboratories, USA, BA-2000) (Brunschwig Chemie, Switzerland, S-2000)	Smooth muscle cell differentiation
Anti-lamin A/C	MAB3211, Millipore, Billerica, USA	1:200	HAM and NHS	Myoblast differentiation
Anti-progerin	Kindly provided by K. Djabali (Department of Dermatology, Colombia University, NY, USA)	1:50	GAR and NGS	Cardiovascular aging
Anti-eNOS	PA1037, Thermo Scientific, Rockford, USA	1:100	GAR and NGS	Susceptibility for aortopathy in BAV
Anti-TGFβ	MO-C40009E, Anogen, ON, Canada	1:1000	HAM and NHS	Susceptibility for aortopathy in BAV
Anti-MMP9	MCA2736, ThermoFisher, Waltham, USA	1:100	HAM and NHS	Susceptibility for aortopathy in BAV
Anti-c-Kit	A4502, Dako, Heverlee, Belgium	1:00	GAR and NGS	Susceptibility for aortopathy in BAV
Anti-pc-Kit	ab62154, Abcam, Cambridge, UK	1:100	GAR and NGS	Susceptibility for aortopathy in BAV
Anti-HIF1α	SC-53546, Santa Cruz Biotechnology, TX, USA	1:500	HAM	Susceptibility for aortopathy in BAV
Anti-FBN1	MAB1919, Millipore, Billerica, Germany	1:100	HAM and NHS	Fibrillin-1 expression

*αSMA* alpha-smooth muscle actin, *SM22α* smooth muscle-22-alpha, *eNOS* endothelial nitric oxide, *TGFβ* transforming growth factor-beta, *MMP9* matrix metalloproteinase-9, *pc-Kit* phosphorylated c-Kit, *HIF1α* hypoxia-inducible-factor-1-alpha, *FBN1* fibrillin-1, *GAR* goat-anti-rabbit-biotin, *NGS* normal goat serum, *HAM* horse-anti-mouse-biotin, *NHS* normal horse serum, *RAM-PO* peroxidase-conjugated rabbit anti-mouse

### Histologic parameters, immunohistochemical analyses, and morphometry

Sections were studied with a Leica BM500 microscope equipped with plan achromatic objectives (Leica Microsystems, Wetzlar, Germany). Cytolytic necrosis and elastic fiber degeneration were defined qualitatively, in alpha-smooth muscle actin (αSMA) and resorcin fuchsin (RF) stained sections, respectively. The cytoplasmatic level of expression of αSMA, smooth muscle-22-alpha (SM22alpha), smoothelin, and MMP-9, intra- and extracellular expression of fibrillin-1, cytoplasmatic and extracellular matrix expression of transforming growth factor-beta (TGFβ), and nuclear expression of lamin A/C, progerin, cleaved-caspase-3, eNOS, pc-Kit, and Hif1α were analyzed on three predetermined locations (left, middle, and right) of every section, that we refer to as ‘microscopic fields’ maintained in evaluation of all staining on sister sections. In each microscopic field, the level of expression was indexed on the three anatomical layers of the aortic wall (tunica intima, media, and adventitia) as 0 (no expression in the respective layer), 2 (expression in less than one third of the layer), 4 (expression in two thirds of the layer), and 6 (expression in the whole layer). To determine the level of lamin A/C, progerin, cleaved-caspase-3, eNOS, pc-Kit and Hif1α expression, the number of positively stained nuclei was counted following previously described methods [6]. All specimens were re-evaluated by an independent, experienced histopathologist who was blinded to the clinical data.

### Statistical analyses

All numerical data are presented as mean ± SD of three microscopic fields on each stained slide. Statistical differences were evaluated with the Mann–Whitney *U* test for comparison between the groups. We also performed a one-, two-, and three-way ANCOVA test to correct for age and gender. Significance was assumed when *p* < 0.05 using SPSS 20.0 software program (SPSS Inc., Chicago, USA). Graphpad software was used to create graphics of statistical analysis.

## Results

### Patient characteristics

Patient characteristics of all five groups are shown in Table 2. The MFS patients were evidently the youngest, followed by the BAV patients. In MFS, male and female were almost equally affected; the BAVs, however, showed a marked male predominance. There was thus a noticeable variance in age and gender distribution in our study. Statistically, both age and gender were not found confounding in our study.

**Table 2:** Clinical characteristics of all patients

Characteristics	TA (*N* = 11)	TAD (*N* = 12)	BA (*N* = 17)	BAD (*N* = 19)	MFS (*N* = 8)
Age (years)	64.5 ± 9.0	72.3 ± 11.2	55.8 ± 9.8	60.7 ± 7.8	34.1 ± 11.8
Males (%)	54.5	33.3	70.1	84.2	62.5
Females (%)	45.5	66.7	29.4	15.8	37.5
Ascending aorta diameter (mean)	^a^	55.0 ± 10.7	36.5 ± 7.4^b^	52.7 ± 6.2	28.4 ± 12.8
Aortic root diameter (mean)	^c^	^c^	^c^	^c^	48.1 ± 3.0
Aortic valve pathology					
No valve pathology	*N* = 11	*N* = 6	*N* = 6	*N* = 3	*N* = 7
Aortic stenosis	*N* = 0	*N* = 1	*N* = 4	*N* = 8	*N* = 0
Aortic regurgitation	*N* = 0	*N* = 5	*N* = 1	*N* = 5	*N* = 1
Aortic stenosis and regurgitation	*N* = 0	*N* = 0	*N* = 5	*N* = 3	*N* = 0

^a^Data unavailable, clinically defined as non-dilated by pathologist

^b^Data unavailable for 5 patients, clinically defined as non-dilated by pathologist

^c^Aortic root diameters unavailable

All MFS patients showed typical aortic root dilation (diameter 48.1 ± 3.0 mm), with a non-dilated ascending aorta (diameter 28.4 ± 12.8 mm). Marked root dilation was not present in the other four groups (TA, BA, TAD, and BAD).

### General histopathologic features in aortic walls of MFS, BAV, and TAV

The ascending aortic wall, consisting of a tunica intima, media, and adventitia, was compared between the MFS, BAV (BA, BAD), and TAV (TA, TAD) groups. The total vessel wall thickness, excluding the highly variable adventitia, was not different between the 5 groups.

**Fig. 1:**
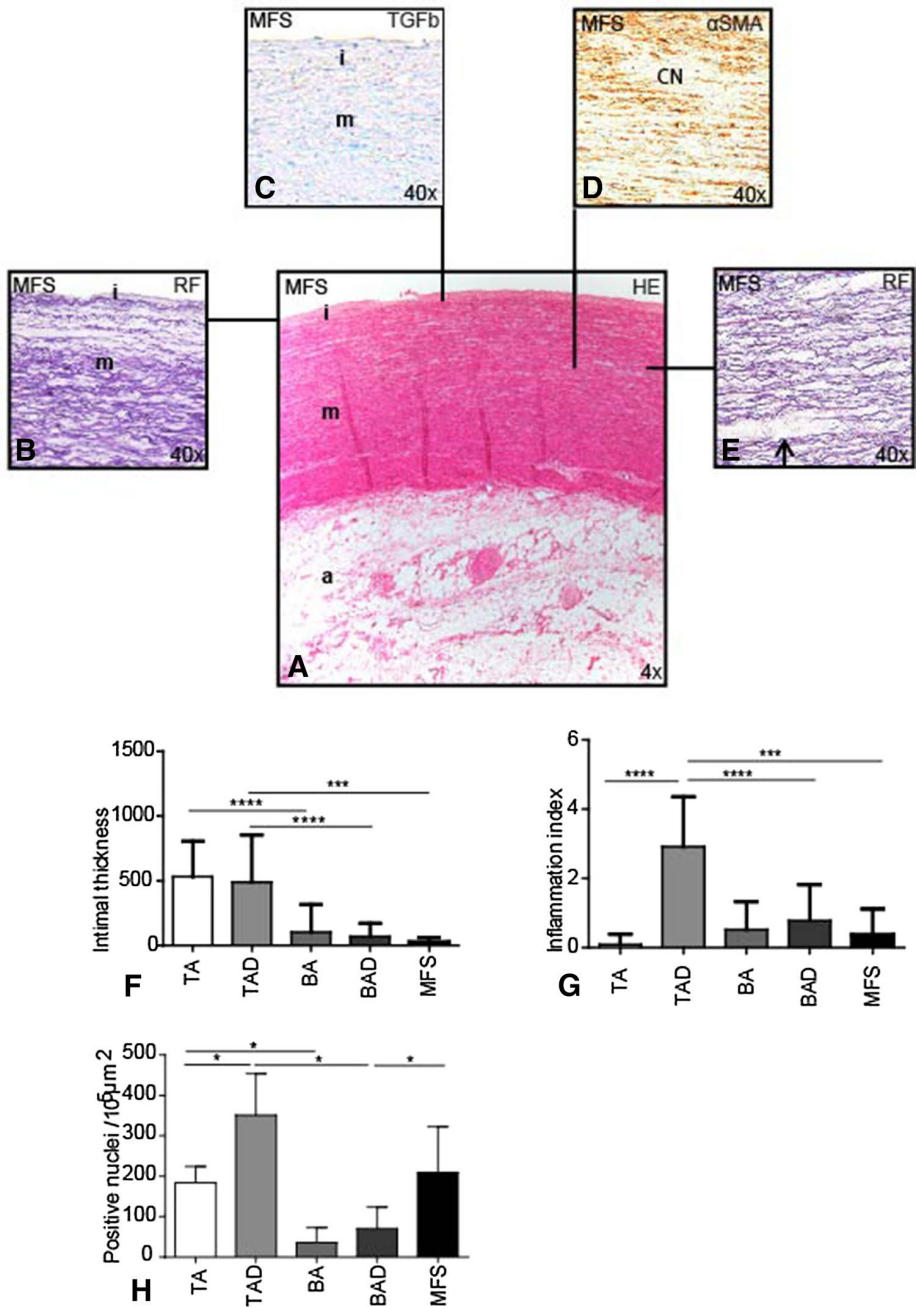
General histologic features in Marfan syndrome. Transverse histologic sections (5 µm) stained with resorcin fuchsin (RF), hematoxylin–eosin (HE) and alpha-smooth muscle actin (αSMA) in Marfan syndrome (MFS). HE-stained overview section (**a**) shows the tunica intima (*i*), which was significantly thinner (**b**, **f**) and lacked TGF-β expression in all MFS (**c**) and BAV patients as compared to the TAV groups, tunica media (*m*) and tunica adventitia (*a*). The aortic media of MFS and TAD showed significant pathology in the media, with more profound cytolytic necrosis (CN) (**d**) and a more fragmented pattern of the elastic lamellae in which the inter-lamellar distance (*arrow*) was enlarged (**e**). Adventitial inflammatory cells were absent in the MFS (**a**) and most outspoken in the TAD group (**g**). Expression of the apoptosis marker cleaved-caspase-3 was significantly elevated in the media of MFS and TAD as compared to the BAD (**h**). Magnification **a** ×4, **b**–**e** ×40. ***p < 0.001, ****p < 0.0001

*Intima* Similar to BAV [5], in MFS, the intima was significantly thinner as compared to the TAV groups (MFS vs TAD *p* < 0.001) (Fig. 1a, b, f). Previously we described that in all specimens from BAV patients, the intima also showed a significantly lower intimal expression of TGFβ as compared to TAV [6]. When analyzed in the MFS group, we found that in this group, the intima also had a very low TGFβ expression (Fig. 1c).

*Media* Of all groups, only the aortic wall of MFS and TAD showed significant pathology in the media, with more profound cytolytic necrosis (Fig. 1d) and a more fragmented pattern of the elastic lamellae in which the inter-lamellar distance was enlarged (Fig. 1e). These signs of pathology were not observed in the TA, BA, and BAD groups (not shown). Expression of the apoptosis marker cleaved-caspase-3 was markedly elevated in the media of MFS and TAD as compared to the BAD (*p* = 0.033 and *p* = 0.0286, respectively) (Fig. 1h).

*Adventitia* The adventitia consisted of loose fibrous tissue containing nerve fibers, fibroblasts, adipocytes, and vasa vasorum, lined by endothelium and VSMCs in all groups. Adventitial inflammatory cells were most outspoken in the TAD group as compared to the MFS and BAD group (*p* < 0.0001, *p* < 0.001, respectively) (Fig. 1a, g).

In conclusion, intimal thickness and lack of adventitial inflammation were similar between BAV and MFS. Aortic media pathology was comparable between the non-dilated MFS and dilated TAV.

### Fibrillin-1, differentiating and mature VSMCs, lamin A/C, progerin, and the pc-Kit pathway

To further understand the pathobiology, we focused on the differentiation state of the aortic wall and the expression of the pc-Kit pathway and fibrillin-1 in all patient groups.

**Fig. 2:**
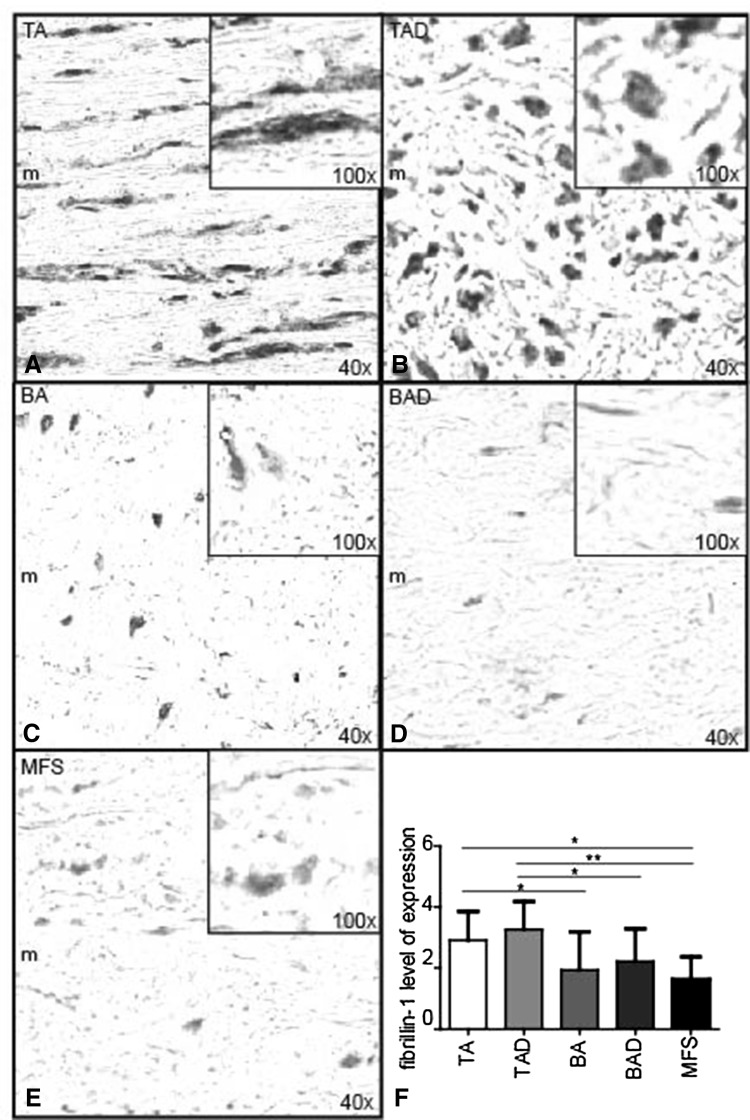
Fibrillin-1 level of expression. Transverse histologic sections (5 µm) stained with fibrillin-1. TA (tricuspidy without dilation), TAD (tricuspidy with dilation), BA (bicuspidy without dilation), BAD (bicuspidy with dilation), MFS (Marfan syndrome without dilation). FBN-1 expression was observed in the aortic media (*m*) of all groups, with each picture (**a**–**e**) showing an inset with magnification ×100 to illustrate the expression on cellular level. The level of expression was significantly lower in all patients with MFS (**e**), BA (**c**), and BAD (**d**) as compared to TA (**a**) and TAD (**b**, **f**). Staining was mainly extracellular in the TA and TAD, whereas in the MFS and BAV, the decreased expression was mainly observed intracellular (cytoplasmic) in the VSMC. Magnification **a**–**e** ×40. *p < 0.05, **p < 0.01

Fibrillin-1 expression was identified in the aortic media of all groups. The total number of cells (positively and negatively stained nuclei) was not different between all investigated specimens from the various groups. The level of expression was, however, significantly lower in all patients with MFS and BAV (BA, BAD) as compared to TAV (TA, TAD) (*p* < 0.05) (Fig. 2a–f). The localization of the staining was different between the groups. In the TAV (TA and TAD), the staining was mainly seen extracellular, whereas in the MFS and BAV besides being decreased, the expression was mainly observed intracellular (cytoplasmic) in the VSMCs (Fig. 2a–e).

**Fig. 3:**
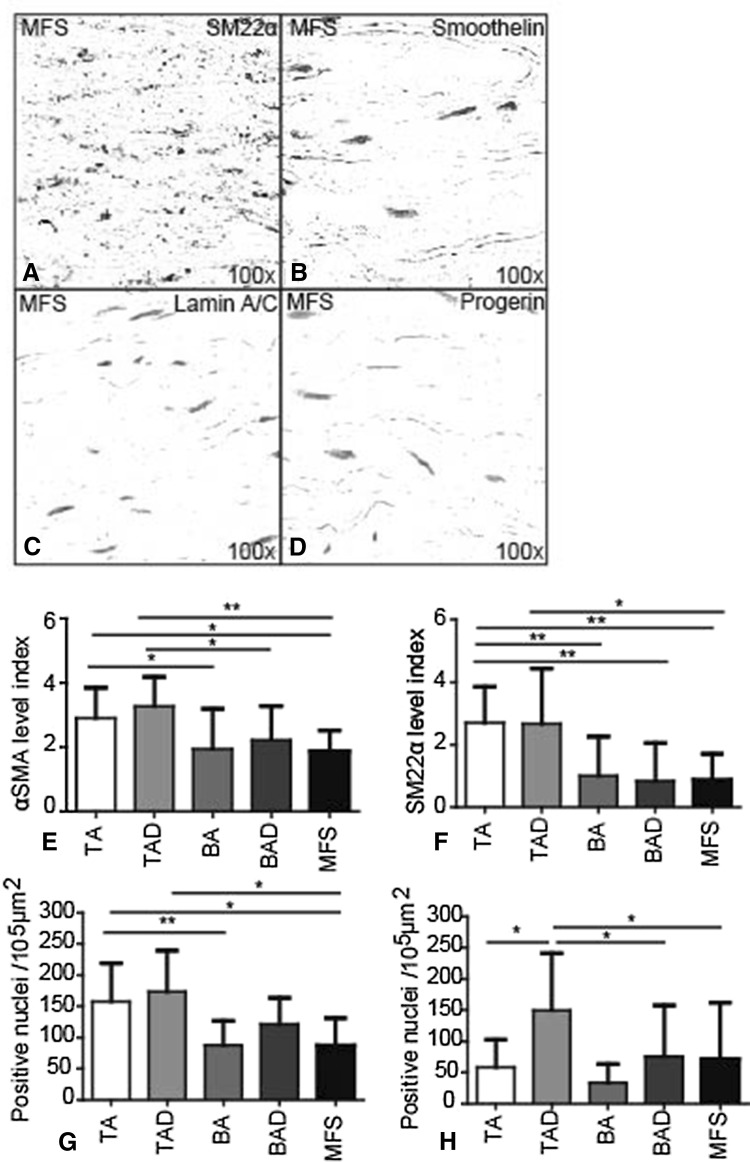
Differentiation markers in Marfan syndrome. Transverse histologic sections (5 µm) stained with smooth muscle 22 alpha (SM22α), smoothelin, lamin A/C, and progerin, markers for differentiation state of VSMCs. SM22α (**a**, **f**), αSMA (**e**), and smoothelin (**b**) expression in MFS was similar to BAD and significantly lower as compared to TAD. Expression of lamin A/C (**c**, **g**) and progerin (**d**, **h**) in MFS was also significantly lower than in TAD. Magnification **a**–**d** ×100. *p < 0.05, **p < 0.01

We further observed that in MFS, the expression of the differentiated smooth muscle cell markers αSMA (Fig. 3e), SM22α (Fig. 3a, f), and smoothelin (Fig. 3b) was similar to the BAVs and significantly lower as compared to the TAVs group. The decreased expression of these markers suggests an immature smooth muscle cell phenotype and less contractility of the vessel wall [21–23]. Expression of lamin A/C (Fig. 3c, g) and progerin (Fig. 3d, h) in MFS was as seen in BAV, being lower as compared to the TAVs. Thus, the aortic wall in MFS shows features of less differentiation comparable with the BAV group. The results are summarized in Table 3.

**Table 3:** Summary of the results

	TA	TAD	MFS	BA	BAD
Intimal thickness	+++	+++	±	±	±
TGFβ intima expression	++	++	−	−	−
Inflammation	±	+++	±	±	±
Cytolytic necrosis	−	+	+	−	−
Apoptosis	++	+++	++	+	+
Elastic lamellae degeneration	−	+	+	−	−
Fibrillin-1	+++	+++	+	+	+
VSMC expression (αSMA, SM22α, smoothelin)	+++	+++	±	±	±
Lamin A/C	+++	+++	+	+	+
Progerin	+	+++	+	+	+

We further investigated markers predictive for ascending aortic dilation in BAV, including pc-Kit [6]. We found that these markers were not expressed in the MFS group (data not shown).

## Discussion

Our study describes an in-depth effort to compare the ascending aortic wall of patients with MFS (non-dilated), BAV (non- and dilated), and TAV (non- and dilated) in one study. A specific aortic wall architecture associated with aortic wall pathology in both MFS and BAV is an extensively discussed, yet controversial, subject. Previous studies compared the aortic wall of MFS and BAV histologically and found that the media were characterized by cytolytic necrosis and elastic fiber degeneration in both diseases [24]. An increased MMP activity and decreased fibrillin-1 in the aortic wall, without comparable reduction in matrix components elastin and collagen, were further noticed [4, 7, 8]. Similarities have thus been noted, though without getting grip on a possible common defect. In this study, we attempted to shed light on intrinsic defects of the aortic wall leading to the observed similarities by comparing the ascending aortic wall of both diseases with each other and with patients with TAV without MFS. As recently several publications focused on a difference in dilation progress after aortic valve repair dependent on whether the diseased aortic valve was stenotic or regurgitant with concomitant root dilation [25, 26]; in the latter, we also paid additional attention to the underlying aortic valve pathology besides structural histopathologic features in the patient groups. In the literature, it is argued that the aortic dilation in the stenotic type is a functional hemodynamic-induced problem, while the aortic wall problem in the root phenotype is genetically determined. Therefore, an isolated aortic valve replacement in stenotic BAV patients is believed to halt the aortic wall dilation. Girdauskas et al. further state that BAV patients with root dilation require a more aggressive surgical approach, being a genetic, connective tissue disorder-like form of aortic disease which is independent of transvalvular flow perturbations [25]. We, however, do not agree on this differentiation into two main groups for a several reasons. Firstly, we postulate that all BAVs are intrinsically a congenital malformation (Grewal et al.) and that an early developmental concomitant defect during embryogenesis contributes to the abnormal maturation of the vascular wall of the ascending aorta independent of a subsequent stenotic or a root phenotype [27]. Secondly, as shown in Table 2, in our study population, aortic valve pathology in non- and dilated BAV and dilated TAV was highly variable showing either stenosis or no stenosis, with or without regurgitation. The dilated BAV and MFS groups even consisted of patients without valve pathology, which indicates that even in the absence of specific aortic valve pathology, aortic complications can occur. We can however not completely refute the influence of hemodynamics on the development of aortic wall complications, as we do not have follow-up data of the operated patients. This is a limitation of our study and an important aspect which should be taken into account in future research.

Histopathologically, in our study, patients in the BAV group did not show marked degenerative features in the aortic media [5], which has also been reported earlier [28, 29]. The media of the ascending aorta in MFS, however, showed resemblance with the TAD with significant cytolytic necrosis, VSMC apoptosis, and degradation of the elastic lamellae, which could predispose the aortic wall for dissection. The MFS group thus showed characteristics of a weak aortic wall, although strikingly the pathologic aorta was not even markedly dilated in the MFS study population as compared to the TAD (Table 2). A role for increased MMP9 production in the occurrence of cytolytic necrosis and extracellular matrix production responsible for increased inter-elastic lamellar distance in TAD and MFS was not supported by our studies. The only marked increase of MMP9 was observed in the non-dilated, susceptible BAV with no marked cytolytic necrosis. So further studies are needed into the role for MMP9 in aortic wall pathology.

To further elucidate the high incidence of aortic wall pathology in MFS and BAV, we studied the level of fibrillin-1 expression. The immature aortic wall of BAV and MFS shared a marked resemblance in the level of expression of fibrillin-1. Decreased *fibrillin*-*1* mRNA and fibrillin-1 protein have been demonstrated before in BAV individuals [8] and can lead to dissociation of VSMCs from medial matrix components [30]. The diagnosis of MFS is however clinical and relies on a set of defined clinical criteria (the Ghent nosology) [2]. The new diagnostic criteria emphasize cardiovascular manifestations of the disorder, in which aortic root aneurysm is one of the prime features. Recently, Pepe et al. [31] identified two *fibrillin*-*1* mutations in a population of eight BAV patients. These mutations had never been detected in MFS patients before. Moreover, these BAV patients did not meet the clinical MFS criteria; therefore, they were not diagnosed with MFS [31]. We can conclude that in the present study and previous studies, a decrease in fibrillin-1 has been reported in four conditions: patients with BAV without a *fibrillin*-*1* mutation [8] (current study), patients with BAV and a *fibrillin*-*1* mutation with [9] or without [31] clinical MFS features, and finally patients with clinically MFS and a *fibrillin*-*1* mutation but no BAV [32].

As fibrillin-1 is produced by VSMCs [33], a significant decrease (of structurally normal) fibrillin-1 is plausible in an aortic wall which constitutes less well-differentiated VSMCs, also without apparent *fibrillin*-*1* mutations. As described in this study, in both MFS and BAV, the aortic wall is primarily less well differentiated which can explain the secondary decrease in the (structurally normal) expression of fibrillin-1. Besides a decreased amount of fibrillin-1 in the aortic wall, in line with earlier research, we found that the distribution and localization of fibrillin-1 was different in the MFS and BAV, with more accumulation within the VSMCs [7, 34, 35], whereas it was mostly seen extracellular in the TAVs. As earlier described by Nataatmadja et al., the few extracellular fibers observed in BAV and MFS were thick and short [7]. Hollister et al. also found a deficiency in the amount of microfibrillar fibers, analyzed immunohistochemically [35], comparable with our results.

As described above, cytolytic necrosis and VSMC apoptosis are observed in both MFS and TAV. However, VSMC apoptosis, which leads to cytolytic necrosis, seems to occur due to a different pathogenetic mechanism in MFS as compared to TAV. In TAV, aging, accompanied by an increased progerin expression, and atherosclerosis cause VSMC apoptosis [5, 36], whereas as we have seen in this study in MFS these features are not apparent. In MFS, the media of the aortic wall contain less well-differentiated VSMCs similar to BAV. Therefore, we hypothesize that there must be a different pathway leading to cytolytic necrosis in MFS, which is on the one hand not related to cardiovascular aging, but on the other hand can also not be associated directly to the immature state of the VSMCs as in BAV where cytolytic necrosis scarce [5, 28].

**Fig. 4:**
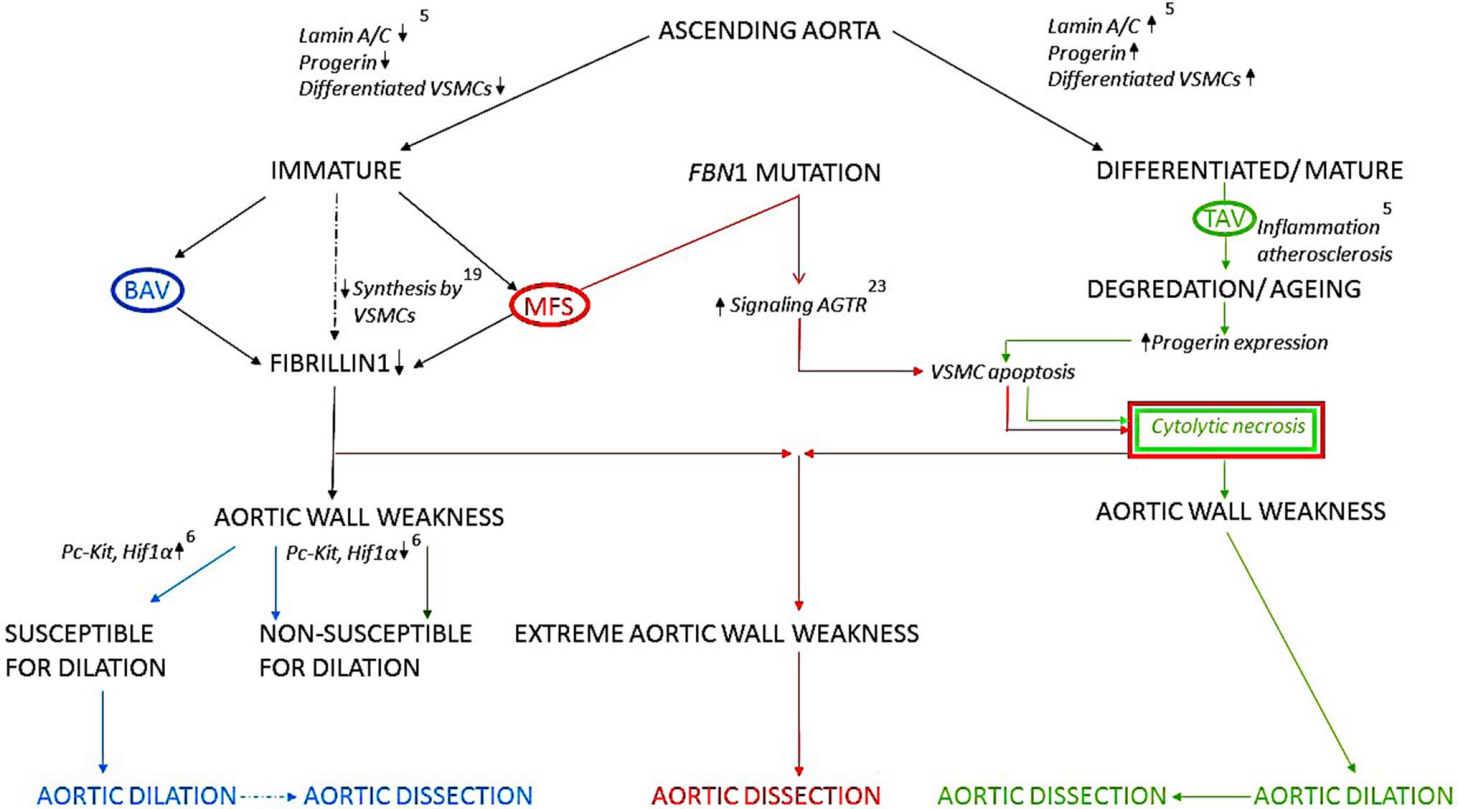
Working hypothesis. Schematic overview of our working hypothesis regarding similarities and differences in aortic wall pathology between bicuspid aortic valve (BAV), tricuspid aortic valve (TAV), and Marfan syndrome (MFS). According to our hypothesis, the ascending aortic wall can be classified as being either mature or less well differentiated. Patients with a TAV have a differentiated/mature vascular wall [differentiated vascular smooth muscle cells (VSMCs) and lamin A/C] in which cardiovascular aging (increased expression of progerin with increased apoptosis, atherosclerosis, and inflammation) accompanies degeneration with features of cytolytic necrosis (CN). This progressive aortic wall pathology in TAV thus leads to a weakened aortic media causing complications as aortic dilation and dissection. In BAV and MFS, weakness of the aorta is however caused by immaturity of the aortic wall (deficient differentiated VSMCs and lamin A/C expression) instead of aging. Fibrillin-1, pivotal for structural stability of the vessel wall, is produced by VSMCs. Immaturity of the vessel thus leads to a quantitative decrease of fibrillin-1 in both BAV and MFS. In MFS, additionally, the *FBN1* (fibrillin-1) mutation leads to VSMC apoptosis through an increased signaling of angiotensin II receptors (AT2 receptor). CN, caused by VSMC apoptosis, in combination with the immature state of the aortic media renders the vascular wall extremely weak, most probably presenting a different pathogenesis of aortic dissection in MFS as compared to the TAV

VSMC apoptosis and subsequent cytolytic necrosis in MFS have earlier been described to occur at a much younger age as compared to the TAV and as a direct consequence of the *fibrillin*-*1* mutation which leads to an increased Angiotensin II (AT2) receptors signaling and subsequently induction of TGFβ signaling [37–39]. We postulate that two different pathologic pathways might thus be distinguished leading to aortic dissections. For clarification, we have schematically provided a working hypothesis (Fig. 4) showing how the various differentiation pathways and the involved gene pathways could lead to the observed histopathological similarities and differences we have seen between the investigated groups. In MFS, there is thus a combination of immaturity of the aortic media and cytolytic necrosis, due to increased VSMC apoptosis, related to increased AT2 receptor signaling [31, 32], rendering the aortic wall very weak. These observations could explain why angiotensin-receptor-blockers (ARBs), as losartan, have been identified as a potentially therapeutic agent to prevent progressive dilation of the ascending aorta in MFS. ARBs reduce the signaling that occurs through both AT receptors, (AT1 and AT2 receptor). Many trials have emerged in recent years investigating the efficacy of ARB’s in human patients with MFS [12–18]. The first results from these studies show reduced dilation of the aortic root in MFS with losartan [17].

A similar positive effect has not been reported in a population of BAV patients who were treated with an angiotensin inhibitor (ACE inhibitor) [19] which can be understood on the basis of our current results. In BAV, medical treatment is not effective because the aortic wall in BAVs is not characterized by apoptosis and cytolytic necrosis. In conclusion, comparison of the aortic wall samples in BAV, MFS, and TAV seems to demonstrate that aortic dissections in MFS might have a different pathogenesis as compared to the TAV, explaining the higher incidence and the younger age of occurrence. These findings could be relevant for understanding why medical treatment to inhibit angiotensin is seemingly successful in MFS and less effective in TAV and BAV patients also being prone for aortic wall pathology.
